# Review of the concept of *Profilicollis* Meyer, 1931 with a description of *Profilicollis rancoensis* n. sp. (Acanthocephala: Polymorphidae) from the freshwater crab, *Aegla abtao* Schmitt, 1942 (Decapoda: Anomura) in Chile, with a key to congeneric species[Fn FN1]

**DOI:** 10.1051/parasite/2023042

**Published:** 2023-10-19

**Authors:** Omar M. Amin, Sara M. Rodríguez, Solinus Farrer, Pablo Fierro, Cristóbal Garcés, Felipe Rivera, Guillermo D’Elía

**Affiliations:** 1 Institute of Parasitic Diseases 11445 E. Via Linda 2-419 Scottsdale AZ 85259 USA; 2 Departamento de Ecología, Facultad de Ciencias, Universidad Católica de la Santísima Concepción Alonso de Ribera 2850 Concepción CP 4030000 Chile; 3 Centro de Investigación en Recursos Naturales y Sustentabilidad (CIRENYS), Universidad Bernardo O’Higgins Avenida Viel 1497 Santiago de Chile CP 8370993 Chile; 4 Department of Biology, Brigham Young University 1114 MLBM Provo UT 84602 USA; 5 Instituto de Ciencias Marinas y Limnológicas, Facultad de Ciencias, Universidad Austral de Chile Campus Isla Teja s/n Valdivia CP 509000 Chile; 6 Núcleo Milenio de Salmones Invasores (INVASAL), Barrio Universitario s/n Concepción CP 403000 Chile; 7 Electron Microscopy Facility, Brigham Young University Provo UT 84602 USA; 8 Instituto de Ciencias Ambientales y Evolutivas, Facultad de Ciencias, Universidad Austral de Chile Campus Isla Teja s/n Valdivia CP 509000 Chile

**Keywords:** *Profilicollis*, North Patagonia, Chile, New species, Molecular characterization COI, 18S

## Abstract

*Profilicollis rancoensis* n. sp. is the tenth species of *Profilicollis* Meyer, 1931 which includes 9 other species mostly known from marine decapod crabs and shore birds. Cystacanths of *P. rancoensis* are described from the dominant freshwater crab *Aegla abtao* in Ranco Lake, Chile and are morphologically distinguished from cystacanths of the 9 other species based on a combination of 4 characters. These are body size, number of proboscis hook rows, number of hooks per row, and length of the largest anterior 2–4 hooks. Male and female cystacanths of *P. rancoensis* are 2.10–3.33 mm long having an ovoid proboscis with 14 rows of 6–7 hooks per row, with the largest anterior 2–4 hooks being 105–110 micrometers long; the anterior trunk has many small spines in 70–80 concentric rings, each with 50–60 spines around them; hook roots are simple, directed posteriorly, about as long as the blades anteriorly with unremarkable anterior manubria; the cephalic ganglion are in mid-receptacle just anterior to the level of the anterior trunk; the lemnisci are long and slender; the testes are in the anterior trunk, posterior trunk, or one in each; the primordia of 2 tubular cement glands are evident; strong bundles of fibers link the anterior and posterior trunk; and the posterior trunk has a corrugated surface cuticula. Molecular analysis (COI and 18S) sequences coincided with the morphology and support its taxonomy. The phylogenetic profile revealed that *P. rancoensis* n. sp. fell into the *Profilicollis* clade. Both sequences showed low genetic variation, and three different haplotypes were found. The new species was more closely related to *P. botulus* (Van Cleave, 1916) Witenberg, 1932 than to other *Profilicollis* species.

## Abstract (fr)

*Profilicollis rancoensis* n. sp. est la dixième espèce de *Profilicollis* Meyer, 1931 qui comprend neuf autres espèces principalement connues de crabes décapodes marins et d’oiseaux de rivage. Les cystacanthes de *P. rancoensis* sont décrits chez le crabe d’eau douce dominant *Aegla abtao* dans le lac Ranco, au Chili et se distinguent morphologiquement des cystacanthes des neuf autres espèces sur la base d’une combinaison de quatre caractères. Il s’agit de la taille du corps, du nombre de rangées de crochets du proboscis, du nombre de crochets par rangée et de la longueur des 2 à 4 crochets antérieurs les plus grands. Les cystacanthes mâles et femelles de *P. rancoensis* mesurent de 2,10 à 3,33 mm de long et ont une trompe ovoïde avec 14 rangées de 6 à 7 crochets par rangée, les 2 à 4 crochets antérieurs les plus grands mesurant 105 à 110 micromètres de long ; le tronc antérieur a de nombreuses petites épines en 70-80 anneaux concentriques chacun avec 50-60 épines ; les racines des crochets sont simples, dirigées vers l’arrière, à peu près aussi longues que les lames vers l’avant avec une manubrie antérieure sans particularité ; les ganglions céphaliques sont au milieu du réceptacle juste en avant du niveau du tronc antérieur ; les lemnisques sont longs et minces ; les testicules sont dans le tronc antérieur, le tronc postérieur ou un dans chacun ; les ébauches des 2 glandes cémentaires tubulaires sont évidentes ; de solides faisceaux de fibres relient le tronc antérieur et postérieur ; le tronc postérieur a une cuticule à surface ondulée. Les séquences d’analyse moléculaire (COI et 18S) coïncidaient avec la morphologie et confirmaient sa taxonomie. Le profil phylogénétique a révélé que *P. rancoensis* n. sp. appartient au clade *Profilicollis*. Les deux séquences ont montré une faible variation génétique et trois haplotypes différents ont été trouvés. La nouvelle espèce était plus proche de *P. botulus* (Van Cleave, 1916) Witenberg, 1932 que des autres espèces de *Profilicollis*.

## Introduction

Cystacanths and adults of *Profilicollis* spp. (Polymorphidae) are common parasites of marine crabs (Decapoda) and shore birds, respectively, mostly along the Pacific and Atlantic coasts of North and South America [25, 50, 51, 54, 56]. Three exceptions include: (i) *Profilicollis formosus* (Schmidt and Kuntz, 1967) Hoklova, 1974 from the freshwater crayfish *Macrobrachium mammillodactylus* (Thallwitz) and the domestic duck *Anas platyrhynchos* Lin. in Taiwan [65], (ii) *Profilicollis major* Lundström, 1942 originally described from Sweden [43] but since found in Maine [50] in scaup, ducks, scoters, and sea otters [62], and (iii) *Profilicollis novaezelandensis* Brockerhoff and Smales, 2002 originally described in New Zealand [13]. *Profilicollis rancoensis* n. sp. represents another exception, like the cystacanths, and has been described from a freshwater South American endemic decapod anomuran *Aegla abtao* Schmitt 1942. A peculiarity of the species of the genus *Aegla*, is that its origin is marine, and because the elevation of Andes was gradual, this allowed the present day Aeglas to adapt to less saline waters [63]. Some of these species were included in dichotomous keys by [12, 33, 52, 59] based on intraspecific variations in proboscis armature and length. The latter key was most comprehensive and Amin [12] proposed synonymizing *Hexaglandula* Petrochenko, 1950 and *Subcorynosoma* Hoklova, 1967 with *Polymorphus* Lühe, 1911.

Most of the studies related to the hosts to species of *Profilicollis* were reported from Pacific South America. Some species of *Profilicollis* may also infect vertebrates other than birds [69]. *Profilicollis altmani* (Perry, 1942) Van Cleave, 1947 can also successfully infect mammals, with potentially serious outcomes that may occasionally represent a public health hazard [46, 57, 69]. In California, United States morbidity and mortality of the southern sea otter were attributed to massive infections with *P. altmani* reaching 8,760 worms per animal, causing intestinal perforations, nutrient depletion, and mortality [46].

Our work contributes to available knowledge of the genus *Profilicollis*, already known to have wide host use and a potential high impact on animal and even human populations. Here, we document the known morphology of this parasite with observations of newly observed structures using scanning electron micrographs and color optical micrographs, provide comparative morphometrical information, analyze the chemistry of hooks and spines for the first time, and produce new molecular analysis of cystacanths of a new acanthocephalan species from Chile.

## Materials and methods

### Collections

Collections of the decapod *Aegla abtao* were made by Lake Ranco at Riñinahue river inlet of Ranco Lake, Los Lagos, Ranco Province, north Patagonia, Chile (40.3331285°S–72.239685°W) at 68 m a.s.l. by hand from logs and large rocks (Fig. Sup.). Once collected, the individuals were sexed, distinguishing females from males by the presence of four pairs of abdominal pleopods, which are used to carry fertilized eggs during the spawning period. An initial collection of 27 crustaceans was made in May 2022, in which we found 7 cystacanths from 4 *Aeglas* (3 males, 1 female). Then in September 2022, we collected 81 crustaceans, in which five individual hosts (4 males, 1 female) were found to be infected with 24 acanthocephalan cystacanths. A third field task was carried out on November 10: we collected 65 crabs, in which four hosts (2 males, 2 females) were found harbouring 6 cystacanths. Another collection of 16 cystacanths and 2 acanthellae was made on December 27, 2022 obtained from 8 hosts (3 males, 5 females), of which 13 specimens and the 2 acanthellae were sent to PCI. We sent a total of 7 specimens to our affiliate laboratory at Brigham Young University, Provo, Utah for SEM and Energy Dispersive X-ray analysis. The remaining 27 specimens (11 males and 16 females) were processed for microscopical studies. A total of 11 specimens were used for sequencing and molecular analysis at Sistematica Lab., Universidad Austral de Chile. A subsequent collection of 259 crabs on four field excursions yielded 53 cystacanths, which were studied.

**Deposited material:** Specimens were deposited in the University of Nebraska’s State Museum’s Harold W. Manter Laboratory (HWML) collection, Lincoln, Nebraska, United States.

### Optical microscope images

Optical microscope images were acquired using a BH2 light Olympus microscope (Olympus Optical Co., Osachi-shibamiya, Okaya, Nagano, Japan) attached to an AmScope 1000 video camera (United Scope LLC, dba AmScope, Irvine, CA, USA), linked to an ASUS laptop.

### Scanning electron microscopy (SEM)

Seven specimens that had been fixed and stored in 70% ethanol were subsequently unsheathed and processed for SEM, following standard methods (Lee, 1992). These methods included drying with a critical point dryer (Tousimis Autosamdri 931.GL; Tousimis, Rockville, MD, USA) and mounting on aluminium stubs using conductive double-sided carbon tape. Samples were coated with an approximately 20 nm layer of 80%–20% gold-palladium using a Quorum Q150T ES magnetron sputtering system (Quorum, Laughton, United Kingdom) equipped with a tilted rotating stage. Samples were placed and observed in a Helios Dual Beam Nanolab 600 Focussed Ion Beam/Scanning Electron Microscope (FIB/SEM) (FEI, Hillsboro, OR, USA). Scanning electron micrographs were acquired using the Helios SEM, with an accelerating voltage of 5 kV, and a probe current of 86 pA, at high vacuum, using and Everhart–Thornley secondary electron detector.

### Focused Ion Beam (FIB) sectioning of hooks

The Helios Dual Beam FIB/SEM, mentioned above, is also equipped with a Focussed Ion Beam (FIB) column with a Ga+ Liquid Metal Ion Source (LMIS). The FIB allows for *in situ* site-specific sectioning of the samples by irradiating precise areas of the sample with a focussed beam of Ga+ ions, which sputter material away and erode the specific areas of the samples. To minimize sample damage due to FIB irradiation, the hooks of the acanthocephalans were centered on the stage while imaging with the SEM. Sectioning time is dependent on the volume of tissue removed, the nature and sensitivity of the exposed tissue, and ion accelerating voltage and current. For this work, the hooks of the acanthocephalans were sectioned using an ion accelerating voltage of 30 kV and a probe current of 2.7 nA in two steps: initial rough removal of bulk material, followed by more precise “cross-section cleaning” that resulted in a smoother exposed surface for SEM imaging and X-ray microanalysis. The hooks were sectioned perpendicular to the long axis at three locations, near the tip of the hook, near the middle of the hook, and near the base. Additional longitudinal sections were prepared near the middle of the hook, which required longer mill times due to the volume of material required for removal.

### Energy Dispersive X-Ray analysis (EDXA)

The Helios Nanolab 600 is equipped with a TEAM Pegasus system with an Octane Plus detector (EDAX, Mahwah, NJ, USA). Energy Dispersive X-Ray (EDX) spectra were collected from the center at each of the exposed surfaces of the sectioned cuts. EDX spectra were collected using an electron accelerating voltage of 15 kV, and a probe current of 1.4 nA. Data collected included images of the sectioned surfaces, images of the displayed spectra, as well as the raw EDX collected data. Relative elemental weight-percentages were generated by the TEAM software. To maintain consistency within the TEAM software, and to ease comparison among various spectra, the following elements list was kept constant among all EDX scans: C, N, O, Na, Mg, Al, P, S, K, Ca, Fe, Au, Pd, and Ga (being aware that Au, Pd, and Ga were process elements used during sample preparation and milling).

### DNA extraction, PCR and sequencing

DNA of parasites, obtained from body cavity of hosts, was extracted from entire individuals (*N* = 11). Genetic comparison and phylogenetic analyses were based on a fragment of 627 bp of the mitochondrial cytochrome oxidase I (COI) gene and 760 bp fragment of the partial 18S rDNA gene. The samples were digested overnight at 55 °C and genomic DNA was isolated using a commercial extraction kit (Wizard^®^ Genomic DNA Purification Kit, Promega, Madison, WI, USA). A fragment of the COI gene was amplified using the primers detailed by Folmer [1622], following the protocol of Amin [67]. The same parasite individuals (*N* = 11) were used to acquire 18 S rDNA sequences. PCR was performed in 30 μL volume reactions containing 2 × red PCR premix (Ampliqon, Odense, Denmark), 20 pmol of each primer, 3 μL of extracted DNA, and primers MGF (5′–GATCGGGGAGGTAGTGACG–3′) and MGR (5′–ACCCACCGAATCAAGAAAGAG–3′). PCR conditions for 18S rDNA were those of Rodríguez et al. [58]. PCR products were analyzed on 1.5% agarose gel and visualized with a UV transluminator. Later, the PCR products were purified and sequenced using an external sequencing service (Macrogen, Inc., Seoul, South Korea). Finally, all new DNA sequences were edited using Codon-Code (Codon Code Aligner, Dedham, MA, USA) and deposited in GenBank (*COI*: OQ417136–OQ417146; *18S*: OQ434741–OQ434748).

### Phylogenetic analysis

The 11 new COI sequences were integrated into a matrix with a representative of each genus of the family Polymorphidae available in GenBank. A total of 41 sequences belonging to the family Polymorphidae were downloaded from GenBank (Table 1) and analyzed. In addition, we used as the outgroup the phylogenetically related species *Plagiorhynchus cylindraceus* and *Centrorhynchus globocaudatis* (Plagiorhynchidae), and *Sphaerirostris lanceoides* (Centrorhynchidae). As such, the analyzed matrix has a total of 55 sequences. A second matrix was made with 15 sequences of COI of each species of the *Profilicollis* available in GenBank and we used *P. cylindraceus* as the outgroup.

**Table 1:** Species of acanthocephalans of the Polymorphidae family, their hosts, locations and GenBank accession number of the sequences used in the phylogenetic analysis. ND = No data available. (–) = Not indicated.

Species	Host	Location	GenBank access COI	GenBank access 18S	References
*Profilicollis rancoensis* n. sp.	*Aegla abtao*	Ranco Lake, Chile	OQ417136–OQ417146	OQ434741–OQ434748	This study
*Profilicollis altmani* (Perry, 1942)	*Leucophaeus modestus*	Valdivia, Chile	KX702245	–	Rodríguez et al. [2017] [56]
*Profilicollis altmani* (Perry, 1942)	*Enhydra lutria*	–	–	AF001838	Near et al. [1998] [49]
*Profilicollis botulus* (Van Cleave, 1916)	*Carcinus maenas*	Wadden Sea, Netherlands	KX279935	–	Goedknegt et al. [2017] [23]
*Profilicollis botulus* (Van Cleave, 1916)	*Somateria mollissima*	Denmark	–	EU267805	García-Varela et al. [2009] [20]
*Profilicollis novaezelandensis* Brockerhoff & Smales, 2002	*Hemigrapsus crenulatus*	New Zealand	MG602475	–	Hay et al. [2018] [27]
*Profilicollis chasmagnathi* (Holcman-Spector, Mane-Garzon & Dei-Cas, 1978)	*Larus dominicanus*	Valdés Peninsula, Argentina	MG859266	–	Lorenti et al. [2018] [41]
*Andracantha* sp.	*Hypomesus japonicus*	Hokkaido, Japan	LC465391	–	Sasaki et al. [2019] [62]
*Arhytmorhynchus brevis* Van Cleave, 1916	*Nycticorax nycticorax*	–	DQ089717	–	García-Varela and Nadler [2006] [17]
*Arhytmorhynchus brevis* Van Cleave, 1916	*Botaurus lentiginosus*	Baja California, Mexico	–	JX442171	García-Varela et al. [2013] [22]
*Arhytmorhynchus frassoni* (Molin, 1858)	*Eudocimus albus*	Sinaloa, Mexico	JX442188	JX442165	García-Varela et al. [2013] [22]
*Bolbosoma balaenae* (Gmelin, 1790)	*Balaenoptera physalus*	Capri Island, Italy	MZ047281	–	Santoro et al. [2021] [60]
*Bolbosoma balaenae* (Gmelin, 1790)	*Nyctiphanes couchii*	Ria de Vigo, Spain	–	JQ040306	Gregori et al. [2012] [26]
*Bolbosoma caenoforme* (Heitz, 1920)	*Salvelinus malma*	Taui Gulf, Asia	KF156891	KF156879	Malyarchuk et al. [2014] [44]
*Bolbosoma turbinella* (Diesing, 1851)	*Paralichthys isosceles*	Rio de Janeiro, Brazil	KU314823	–	Not published
*Bolbosoma turbinella* (Diesing, 1851)	*Eschrichtius robustus*	Monterrey Bay, USA	–	JX442166	García-Varela et al. [2013] [22]
*Bolbosoma vasculosum* (Rudolphi, 1819) Porta, 1908	*Lepturacanthus savala*	Java, Indonesia	–	JX014225	Verweyen et al. [2011] [77]
*Bolbosoma* sp.	*Homo sapiens*	Japan	LC377776	–	Kaito et al. [2019] [34]
*Bolbosoma* sp.	*Callorhinus ursinus*	Alaska, USA	JX442190	JX442167	García-Varela et al. [2013] [22]
*Corynosoma bullosum*	*Mirouga leonina*	King George Island, Antarctic Peninsula	OP142751	–	Not published
*Corynosma bullosum*	*Mirounga leonina*	King George Island, Antarctic Peninsula	–	OQ192986	Not published
*Corynosoma australe* Johnston, 1937	*Stenella clymene*	Argentina	MW724483	–	García-Varela et al. [2013] [22, 30]
*Corynosoma australe* Johnston, 1937	*Phocarctos hookeri*	New Zealand	–	JX442168	García-Varela et al. [2013] [22]
*Corynosoma enhydri* Morozov, 1940	*Enhydra lutris*	–	DQ089719	–	García-Varela and Nadler [2006] [17]
*Corynosoma enhydri* Morozov, 1940	*Enhydra lutris*	–	–	AF001837	Near et al. [1998] [49]
*Corynosoma hannae* Zdzitowiecki, 1984	*Colistium guntheri*	Otago, New Zealand	KY909263	–	Anglade and Randhawa [2018] [12]
*Corynosoma magdaleni* Montreuil, 1958	*Phoca vitulina*	North Baltic Sea, Germany	MF078642	–	Not published
*Corynosoma magdaleni* Montreuil, 1958	*Phoca hispida saimensis*	Lake Saimaa, Finland	–	EU267803	García-Varela et al. [2009] [20]
*Corynosoma nortmeri* Waindoka, Lehnert, Siebert, Pawliczka & Strube, 2018	*Phoca vitulina*	North Baltic Sea, Germany	MF001278	–	Waindok et al. [2018] [78]
*Corynosoma obtuscens* Lincicome, 1943	*Callorhinus ursinus*	Alaska, USA	JX442192	JX442169	García-Varela et al. [2013] [22]
*Corynosoma semerme* (Forssell, 1904) Lühe, 1911	*Phoca largha*	Hokkaido, Japan	LC465313	–	Sasaki et al. [2019] [62]
*Corynosoma strumosum* (Rudolphi, 1802) Lühe, 1904	*Neophocaena phocanoides*	Hokkaido, Japan	LC465402	–	Sasaki et al. [2019] [62]
*Corynosoma strumosum* (Rudolphi, 1802) Lühe, 1904	*Phoca vitulina*	Monterrey Bay, USA	–	EU267804	García-Varela et al. [2009] [20]
*Corynosoma validum* Van Cleave, 1953	*Callorhinus ursinus*	Alaska, USA	MK119252	–	Lisitsyna et al. [2019] [40]
*Corynosoma validum* Van Cleave, 1953	*Callorhinus ursinus*	Alaska, USA	–	JX442170	García-Varela et al. [2013] [22]
*Hexaglandula corynosoma* (Travassos, 1915)	*Nyctanassa violacea*	Mexico	EU189488	EU267808	García-Varela et al. [2009] [20]
*Ibirhynchus dimorpha* (Schmidt, 1973)	*Eudocimus albus*	Gulf of Mexico	GQ981438	GQ981436	García-Varela et al. [2011] [21]
*Polymorphus magnus* Skrjabin, 1913	*Larus schistisagus*	Russia	OL689013	–	Not published
*Polymorphus minutus* (Zeder, 1800) Lühe, 1911	*Gammarus pulex*	Dijon, France	EF467865	–	García-Varela and Ponce de León [2008] [18]
*Polymorphus minutus* (Zeder, 1800) Lühe, 1911	*Gammarus pulex*	Dijon, France	–	EU267806	García-Varela et al. [2009] [20]
*Polymorphus obtusus* Van Cleave, 1918	*Aythya affinis*	California, Mexico	JX442195	JX442172	García-Varela et al. [2013] [22]
*Polymorphus phippsi* Kostylev, 1922	*Gammarus setosus*	Russia	OL676687	–	Not published
*Polymorphus trochus* Van Cleave, 1945	*Fulica americana*	Sinaloa, Mexico	JX442196	JX442173	García-Varela et al. [2013] [22]
*Polymorphus* sp.	*Anas platyrhynchos*	Austria	MT184813	–	Not Published
*Polymorphus* sp.	*Anas platyrhynchos*	–	–	AF064815	García-Varela et al. [2000] [19]
*Polymorphus* sp.	*Oligosarcus jenynsii*	Arroyo Grande, Argentina	MT580125	–	Levy et al. [2020] [39]
*Pseudocorynosoma anatarium* (Van Cleave, 1945) Aznar, Perez-Ponce de Leon & Raga, 2006	*Bucephala albeola*	Mexico	KX688148	EU267801	García-Varela et al. [2009] [20]
*Pseudocorynosoma constrictum* (Van Cleave, 1918) Aznar, Perez-Ponce de Leon & Raga, 2006	*Anas clypeata*	Mexico	EU267820	EU267800	García-Varela et al. [2009] [20]
*Pseudocorynosoma tepehuanesi* García-Varela, Hernández-Orts & Pinacho-Pinacho, 2017	*Oxyura jamaicensis*	Mexico	KX688139	JX442175	García-Varela et al. [2013] [22]
*Southwellina hispida* (Van Cleave, 1925)	*Egretta garzetta*	Tabasco, Mexico	EF467868	–	García-Varela and Ponce de León [2008] [18]
*Southwellina hispida* (Van Cleave, 1925)	ND	Baltic Sea, Finland	–	EU267809	García-Varela et al. [2009] [20]
*Tenuisoma tarapungi* Presswell, Bennett & Smales, 2020	*Chroicocephalus novaehollandiae*	New Zealand	MN688202	–	Presswell et al. [2020] [53]
*Centrorhynchus globocaudatus* (Zeder, 1800)	*Buteo buteo*	Italy	MT992255	–	Amin et al. [2022] [9]
*Sphaeristrosis lanceoides* (Petrochenko, 1949)	*Bufo gargarizans Cantor*	Yuyao, China	MG931942	–	Kang and Liang [2018] [36]
*Plagiorhynchus cylindraceus* (Goeze, 1782)	*Atelerix algirus*	Balearic Islands, Spain	MK300542	–	Not Published
*Plagiorhynchus cylindraceus* (Goeze, 1782)	*Armadillium vulgare*	ND	–	AF001839	Near et al. [1998] [49]

Sample size for the partial 18S rDNA gene was slightly smaller than for COI, including eight sequences of the new species of *Profilicollis* and 27 sequences of members of the family Polymorphidae available in GenBank (Table 1). One sequence of *Plagiorhynchus cylindraceus* (Plagiorhynchidae family) was used as the outgroup (Table 1). As such, the analyzed 18S rDNA matrix had a total of 36 sequences. As with COI, a second matrix was created with 10 sequences of 18S rDNA of each species of the genus *Profilicollis* available in GenBank. One sequence of *P. cylindraceus* was used as the outgroup.

Each gene matrix was aligned separately using MAFFT v.7 software [37], allowing the program to choose the alignment strategy (L-ins-i). Four models were carried out: two to analyze the family Polymorphidae, and the other two to analyze the genus *Profilicollis*. To select the best-fitting model of molecular evolution, we used the proposed model tool in the IQ-Tree v1.6.12 program [35], which selected K3Pu+F+I+G4 and TIM3e+I+G4 for the COI and 18S matrix of the family Polymorphidae, respectively and HKY+F+G4 for COI and TPM3+F for 18S for the matrix of the genus *Profilicollis*. We used the maximum-likelihood (ML) approach implemented in IQ-Tree v1.6.12 [72]. For each matrix, we conducted the following analyses: a) Five runs modifying the strength of the perturbation parameter from 0.5 (default value) to 0.3, b) Five runs using the default value (0.5) for the strength of the perturbation parameter, and c) Five runs modifying the strength of the perturbation parameter from 0.5 (default value) to 0.7.

In all cases, the number of unsuccessful interactions to stop parameter (−*n*stop) value was changed from 100 (default value) to 1000. Trees with the highest likelihood score were chosen (family Polymorphidae: COI = log-likelihood: −11404.169, −pers 0.3 and −*n*stop 1000; 18S = log-likelihood: −6647.679, −pers 0.3 and −*n*stop 1000) (genus *Profilicollis*: COI = log-likelihood: −2599.057, −pers 0.3 and −*n*stop 1000; 18S = log-likelihood: −3574.388, −pers 0.5 and −*n*stop 1000). Support for the nodes was evaluated using two approaches: the aBayes test [11] and the ultrafast bootstrap procedure using 1000 replicates [31]. Finally, observed genetic *p*-distances (*p*) between haplotype and sample pairs were calculated in MEGA 7 [68].

## Results

Of a total of 259 crabs examined (*A. abtao*), 21 were parasitized (prevalence [P] = 8.10%), yielded a total of 53 cystacanths. All were from the body cavity (mean abundance [MA: amount of parasites /number of hosts] = 0.2; mean intensity [MI: amount of parasites/number of infected hosts] = 2.5). The number of cystacanths per freshwater crab ranged from 1 to 11. Of the total number of crabs caught, 193 were male and 66 were female. The prevalence (P) in males was 4.2% *vs*. (P) in females 3.8%. The MA in males was 0.13, and 0.30 in the female cystacanths *per* host. The MI values were 3 and 2 cystacanths *per* parasitized host, respectively.

The microscopical study provided considerable information depicted in the line drawings (Figs. 1 –6). The scanning electron microscopy studies produced considerable new detail adding more information not readily available by the optical microscopy study. Our descriptive account of cystacanths is based on 27 cystacanths (11 males and 16 females) and 2 acanthellae measured and studied with optical microscopy from whole mounts (Figs. 1–6) and 7 cystacanths studied with SEM (Figs. 7–22).

**Figures 1–4:**
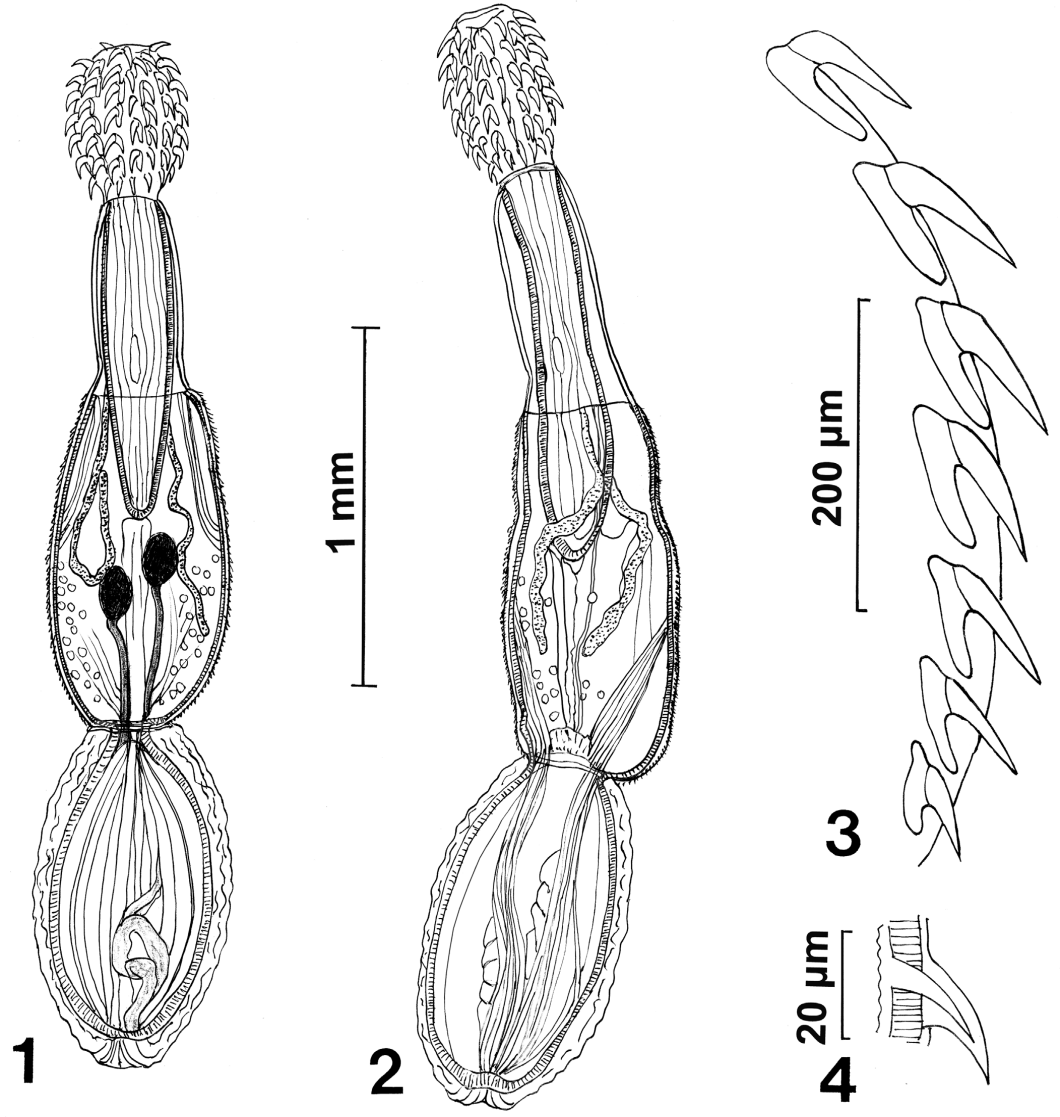
Cystacanths of *Profilicollis rancoensis* n. sp. from the freshwater crab, *Aegla abtao* Schmitt (Decapoda) in Chile. **(1, 2)** A male and female cystacanth. **(3)** One longitudinal row of proboscis hooks showing the variable root structures with different degrees of manubriation. **(4)** Profile of a trunk spine.

**Figures 5, 6.**
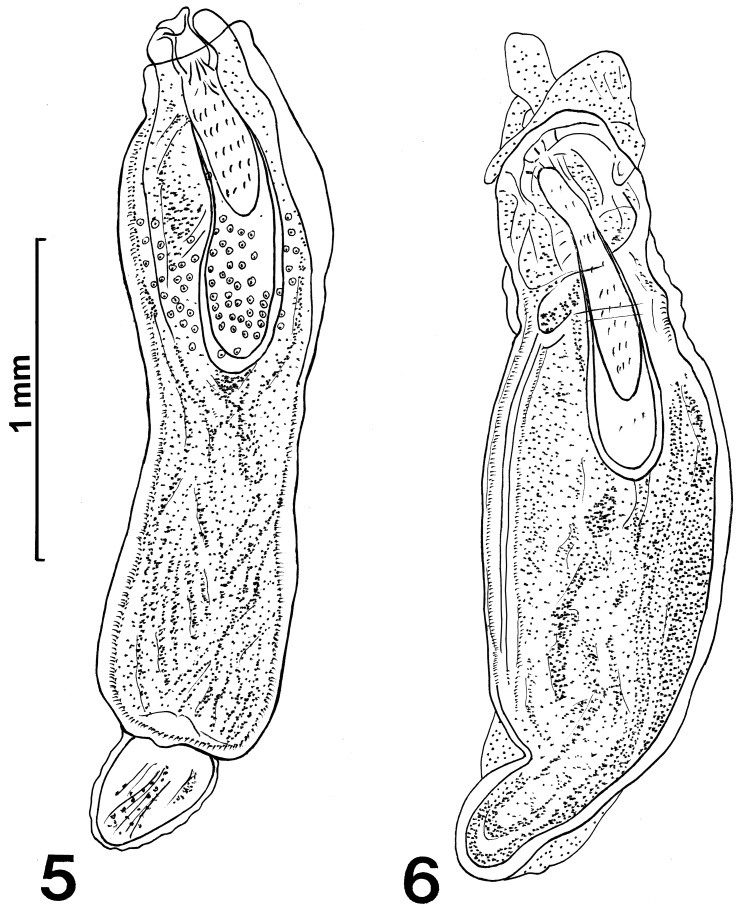
Two late acanthellae at slightly different ages.

**Figures 7–10:**
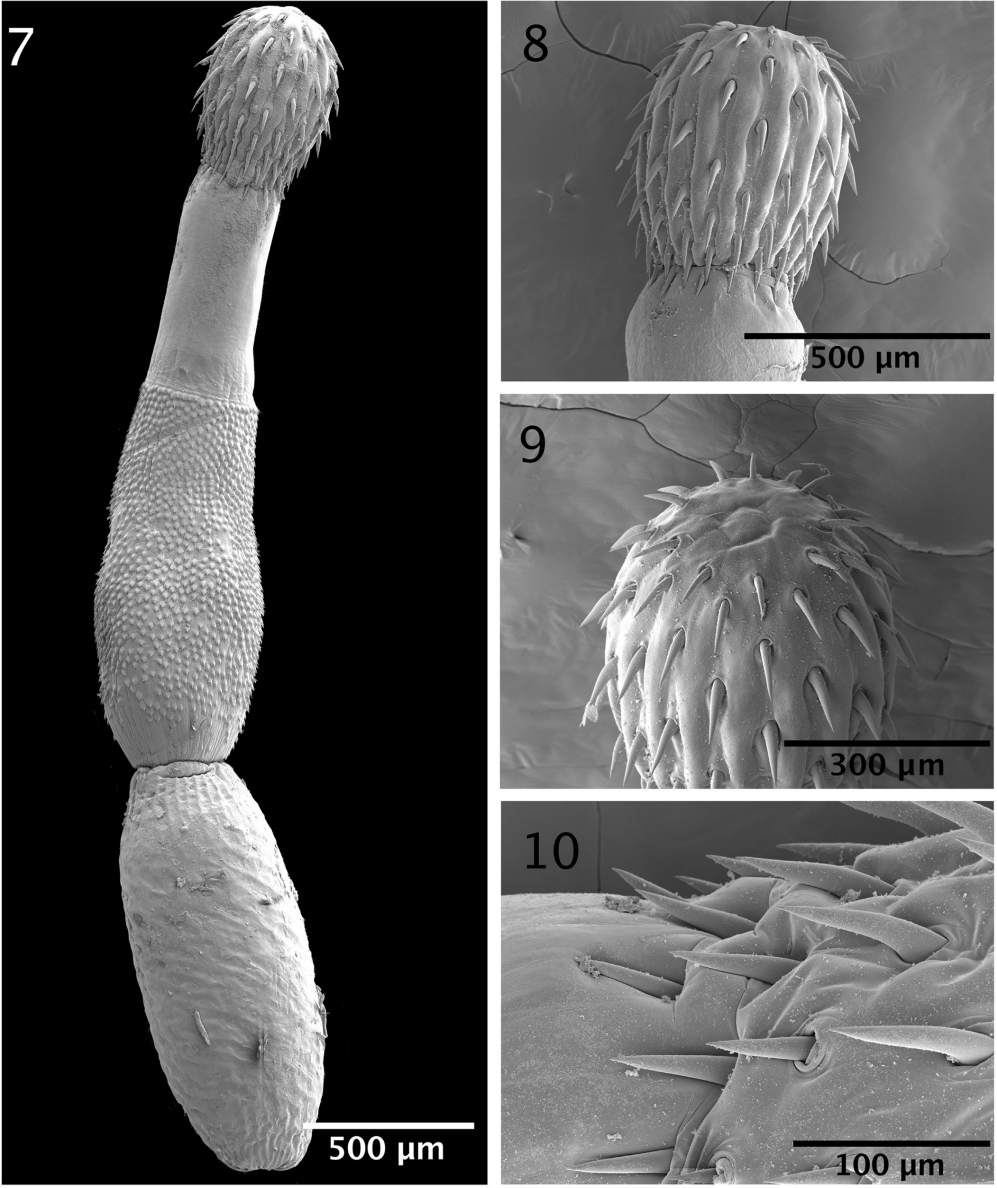
SEM of cystacanths of *Profilicollis rancoensis* n. sp. from *Aegla abtao* in Ranco Lake, northern Patagonia, Chile. **(7)** A montage of a whole specimen after excystation. **(8)** A lateral view of a proboscis showing the organization of hooks. Note that some hooks in the posterior circle appear to emanate from the neck. **(9)** A semi-apical view of a proboscis showing the smaller apical hooks and the external appearance of possible apical organ. **(10)** Posterior circles of hooks showing the insertion of some posterior-most hooks outside of the actual proboscis in the neck.

**Figures 11–16:**
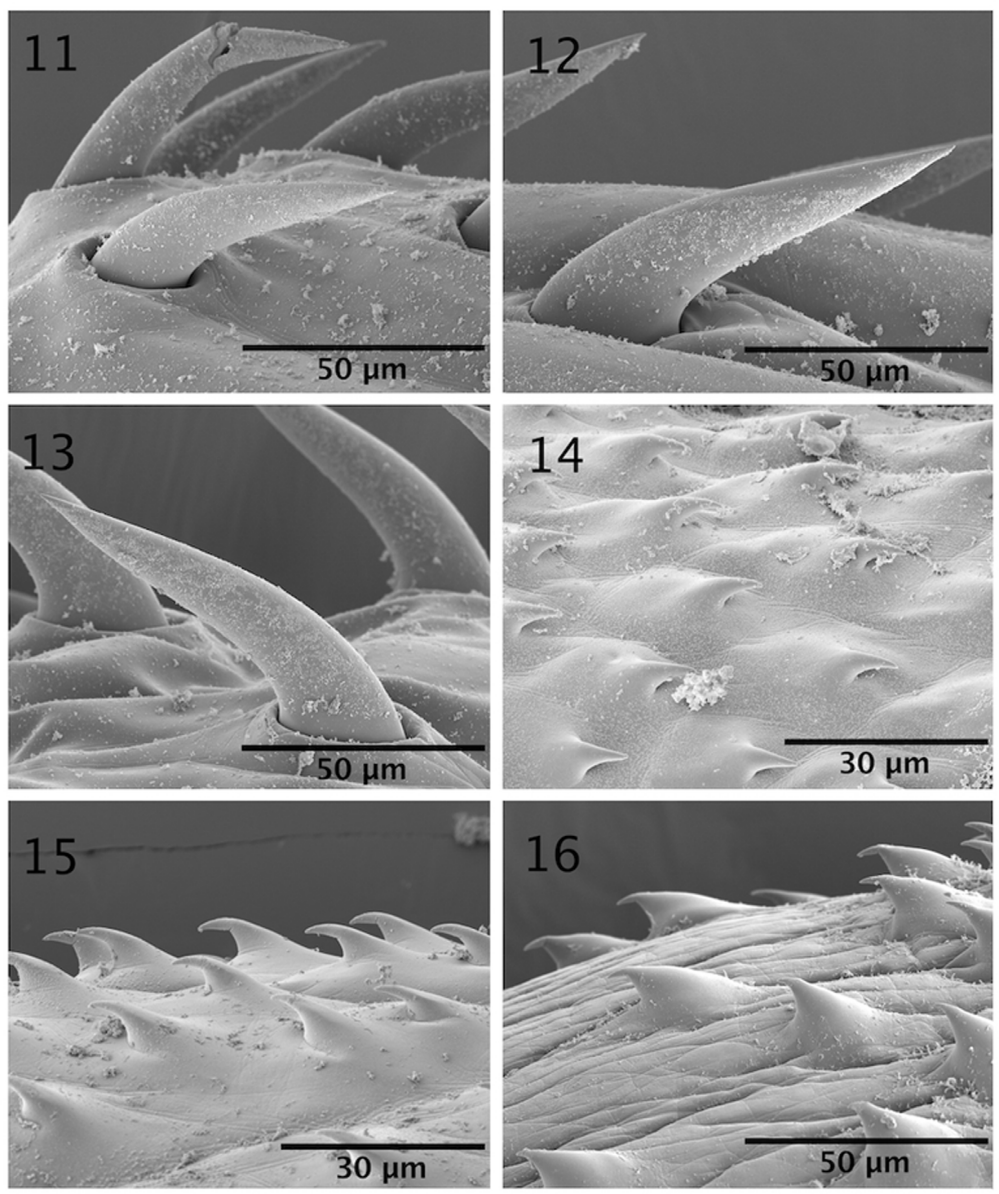
SEM of cystacanths of *Profilicollis rancoensis* n. sp. from *Aegla abtao* in Ranco Lake, Valdivia, Chile. **(11–13)** Anterior, middle and posterior hooks in profile. Note differences in size, shape, and curvature. **(14**–**16)** Anterior, middle and posterior spines; their shape is variable.

**Figures 17–22:**
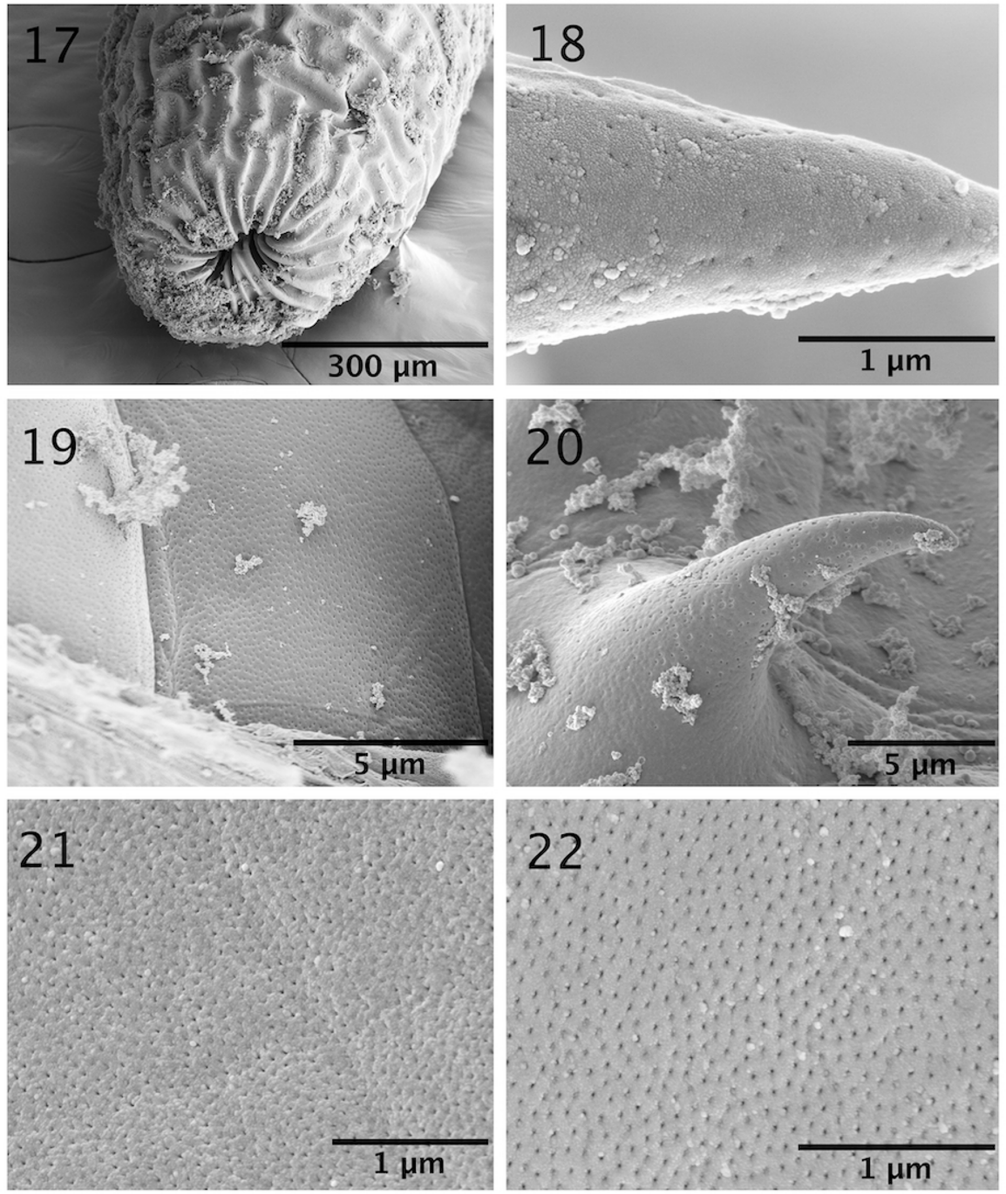
SEM of cystacanths of *Profilicollis rancoensis* n. sp. from *Aegla abtao* in Ranco Lake, Valdivia, Chile. **(17)** The posterior tip of the corrugated posterior trunk (in red). The drying process may have made the corrugation more evident. Manifestations of the various size and distribution of micropores at the tip of a hook **(18)**, proboscis base **(19)**, trunk spine **(20)**, and middle and posterior trunk locations **(21, 22)**.

Primary diagnostic characters of *P. rancoensis* cystacanths including trunk length and proboscis armature (Table 2) are compared with those of 9 other species of *Profilicollis*: adults of *P. major* from Europe and Maine [58, 68] in scaup, ducks, scoters, and sea otters [62], and *Profilicollis novaezelandensis* from New Zealand in oystercatcher [16]. Morphometric details of male and female cystacanths of *P. rancoensis* are noted in Table 3.

**Table 2:** Diagnostic characteristics of *Profilicollis rancoensis* n. sp. from the freshwater crab *Aegla abtao* Schmitt, 1942 (Decapoda) in Chile compared to the 9 other species in the genus.

Species of *Profilicollis*	Trunk length (mm)		Proboscis hooks		Max. hook L. (μm)	Lemnisci	Type or primary hosts		Type locality and/or distribution

	Adults MM & FF	Cysta-canths	Rows	Hooks per row	Hooks 2–4 length	Length vs. receptacle	Intermediate host (mostly Decapoda)	Final host (usually birds)	By country and region
*P. rancoensis* n. sp.	–	2.10–3.33	14–15	6–7	105–110	Very long, slender	*Aegla abtao* (freshwater)	Unknown	Ranco Lake, Valdivia, Chile
*P. altmani* (Perry, 1942) Van Cleave, 1947	4.5–14.0	4.0–7.8 Post. trunk with tail	21–35	9–16	51–70	Plump, equal to receptacle	*Emerita analoga*	*Larus* spp., *Melanitta* spp., *Marilla affinis*, etc. & mammals	Pacific & Atlantic North & South America
*P. antarcticus* Zdzitowiecki 1985	14.1–21.1	3.05–4.53	18–22	7–9, 6–8	71–80	Cylinrical, thick, longer than recept.	*Hemigrapsus crenulatus; Helice crassa*	*Chionis alba* & *Larus dominicanus*	Chile & New Zealand; South Shetland Islands
*P. arcticus* Van Cleave, 1920) Meyer, 1932	16.0–25.0	–	22	7–8	89–118	Pyriform, little longer than recept.	–	*Somateria spectabilis*; *S. nigra*	North America, NW Canada & Russia
*P. botulus* (Van Cleave, 1916) Witenberg, 1932	13.0– 22.0	1.8–2.6	15–20	7–8	80–96; 76–87	Plump-bulbous, ca. equal to receptacle	*Carcinus maenas*; *Hemigrapsus oregonensis*; *Hyas araneus*	*Somateria mollissima* & *Bucephala clangula*, etc.	North America; Scotland & Eurasia
*P. chasmagnathi* (Holcman-Spector, Maňé-Garzón, Dei-Cas, 1978) Amin, 1992	2.5–28.6	1.5–4.1	16–22	7–9	83–91 Post. hooks longer	Slender, little longer than receptacle	*Chasmagnathus granulata*, *Cryptograpsus, angulatus*, *C. altimanus*	*Larus atlanticus, L. dominicanus*	Montevideo, Uruguay & Mar Chiquita, Argentina; SW Atlantic coast
*P. formosus* (Schmidt & Kuntz, 1967) Hoklova, 1974	13.0–18.5	2.0	12–15	7–9	95–117	Long, flat, widest at middle	*Macrobrachium mammillodactylus* (freshwater crayfish)	*Anas platyrhynchos*	Taiwan
*P. major* Lundström, 1942	16.0–40	–	16–20	7–10	92–108	Long, widest at middle	*Cancer irroratus* off Eastern coast of N. America	*Bucephala* spp., *Aythya affinis*, *Melanitta deglandii*, *Enhydra lutris nereis*	Sweden, Europe, Maine (USA)
*P. novaezelandensis* Brockerhoff and Smales, 2002	4.4–22.0	3.5–4.3	14–16	7–8	85–110 hook tips recurved	Long, thick, cylindrical	*Hemigrapsus crenulatus*; *Macrophthalmus hirtips*; *Helix crassa*	*Haematopus ostralegus finschi*; *Limosa lapponica*	Avon Heathcote Estuary, New Zealand & Golden Bay, South Island
*P. sphaerocephalus* (Bremser in Rudolphi, 1819) Van Cleave, 1947	9.0–22.0	2.3–3.5, ant. trunk cylindrical	17–21	7–8	69–73 (150 ?)	Long, cylindrical, slender	*Paragrapsus* spp.; *Brachynotus spinosus* & *Cyclograpsus granulosus, Cherax*	*Larus dominicanus, L. novaehollandiiae Haematopus* spp.	Brazil, Montevideo, South Australia, Tasmania

**Table 3:** Comparative morphometrics of male and female cystacanths of *Profilicollis rancoensis* n. sp. from *Aegla abtao* in Ranco Lake, Valdivia, Chile.

Character	Males (*n* = 11)	Females (*n* = 16)
Total trunk length × width (mm)	*2.10–3.10 (2.59) × 0.50–0.67 (0.56)	2.40–3.32 (2.72) × 0.27–0.67 (0.54)
Ant. trunk length × width (mm)	0.75–1.12 (1.00) × 0.35–0.60 (0.51)	0.77–1.25 (1.06) × 0.40–0.67 (0.55)
Post. trunk length × width (mm)	1.00–1.37 (1.12) × 0.50–0.70 (0.57)	1.02–1.22 (0.98) × 0.52–0.62 (0.57)
Neck length × width post. (mm)	0.40–0.80 (0.55) × 0.22–0.37 (0.30)	0.32–0.70 (55) × 0.27–0.35 (0.31)
Proboscis length × width (μm)	325–550 (471) × 225–370 (303)	375–625 (469) × 291–450 (331)
Receptacle length × width (mm)	0.85–1.20 (1.05) × 0.20–0.40 (0.28)	1.02–1.25 (1.13) × 0.17–0.32 (0.28)
Ant. testis length × width (μm)	135–239 (189) × 72–114 (88)	N/A
Post. testis length × width (μm)	146–270 (179) × 83–104 (95)	N/A
Lemnisci length × width (μm)	572–936 (832) × 62–135 (90)	697–1092 (1006) × 52–162 (118)
Hook rows × hooks per row	14–15 × 6–7	14–15 × 6–7
Hook 1 length × root 1 length (μm)	65–77 (72) × 48–52 (50)	70–90 (79) × 60–65 (62)
Hook 2 length × root 2 length (μm)	75–87 (81) × 62–70 (65)	80–105 (91) × 78–82 (80)
Hook 3 length × root 3 length (μm)	88–97 (91) × 50–77 (66)	75–107 (95) × 66–73 (70)
Hook 4 length × root 4 length (μm)	80–107 (91) × 62–67 (65)	88–110 (96) × 57–65 (61)
Hook 5 length × root 5 length (μm)	70–95 (85) × 52–57 (55)	88–100 (93) × 62–75 (68)
Hook 6 length × root 6 length (μm)	70–100 (84) × 40–45 (43)	75–102 (91) × 57–62 (60)
Hook 7 length × root 7 length (μm)	50–77 (68) × 33–44 (40)	58–82 (72) × 40–60 (48)
Spine circles × spines per circle	69–80 (74) × 35–60 (48)	66–81 (74) × 50–60 (54)
Spine length × diameter (μm)	25–30 (27) × 7–15 (10)	20–30 (26) × 7–15 (10)

* Numbers shown are ranges followed by the mean in parentheses ().

### *Profilicollis rancoensis* n. sp.

urn:lsid:zoobank.org:act:8821DEF9-2BFC-49E2-BB9E-5D0A2718F688

Host: The freshwater Pancora crab *Aegla abtao* Schmitt, 1942 (Crustacea: Decapoda)

Type locality: Riñinahue river inlet of Ranco Lake, Los Lagos, Chile (40.3331285°S–72.239685°W)

Site of infection: Body cavity cephalothorax

Type specimens: Cystacanths & 1 acanthella in HWML Parasitology Collection no. (HWML 216948–HWML 216950)

Etymology: The new species is named for the collection locality of the decapod intermediate host.

#### Remarks on cystacanths

Cystacanths of *P. rancoensis* n. sp. are distinguished from cystacanths of all other species of *Profilicollis* by a combination of the following characters: “In freshwater decapod, *Aegla abtao* from Chile. Proboscis with 14–15 rows of 6–7 hooks each reaching 105–110 μm. All hooks with slight anterior manubrial and strong simple roots; basal hook with small simple root and anterior manubrium. Lemnisci long, slender.” With the exception of *P. formosus* whose cystacanth develop in the freshwater knob-tooth prawn *Macrobrachium mammillodactylus* Thallwitz in Taiwan [65], all other species of *Profilicollis* utilize marine decapods as intermediate hosts (Table 2). A special note needs to address *P. arcticus* whose cystacanth hosts are yet to be identified. The adults of *P. arcticus* have been described and reported from sea ducks, *i.e.*, king eider *Somateria spectabilis* Linn. and Pacific eider *S. nigra* Gray from the arctic coasts of North America, NW Canada, and Russia. The marine distribution of these shore ducks suggests that the intermediate hosts of cystacanths of *P. arcticus* may well be marine decapods as has been suggested by Nickol *et al.*’s [50] characterization of *Profilicollis*. In addition to being the only species of *Profilicollis* using a freshwater decapod as intermediate host, *P. rancoensis* is further distinguished from all other species of *Profilicollis* by the following key. The key includes only one major intermediate host of each species as there may be many in some species. Note that hooks attain their maximum size in cystacanths, which is usually comparable to that in adults of the same species. The detailed coverage in Table 2 does not include all the characters selected for the key. Diagnostic characters used were chosen among features common to both sexes of cystacanths and adults, especially proboscis armature. Reference to simple roots indicates normal posteriorly directed roots.

#### Key to cystacanths of species of *Profilicollis*

1. In freshwater prawns, *Macrobrachium mammillodactylus* from Taiwan. Proboscis with 12–15 rows of 7–9 hooks, each reaching 95–117 μm. Anterior hooks with strong simple roots; posterior hooks rootless. Lemnisci long, flat. ….………………………………………………………*Profilicollis formosus*

– In decapod crustaceans elsewhere. Proboscis armature and lemnisci variable………………………….…2

2. In freshwater decapod, *Aegla abtao* from Chile. Proboscis with 14–15 rows of 6–7 hooks, each reaching 105–110 μm. All hooks with slight anterior manubrial and strong simple roots; basal hook with small simple root and anterior manubrium. Lemnisci long, slender…………………….…..…….*Profilicollis rancoensis* n. sp.

– In marine decapods elsewhere. Proboscis armature and lemnisci variable………………………………….3

3. In *Emerita analoga* from Pacific and Atlantic North and South America. Posterior trunk with tail. Proboscis with 21–35 rows of 9–16 hooks, each reaching 51–70 μm. All hooks rooted with prominent anterior manubria. Lemnisci plump, usually as long as receptacle……………………………………………*Profilicollis altmani*

– Posterior trunk without tail. Proboscis with 14–22 rows of 6–10 hooks each. ………………………………..4

4. In *Paragrapsus* spp. from Brazil, Australia and Tasmania. Anterior trunk cylindrical. Proboscis with 17–21 rows of 7–8 hooks, each reaching 69–73 μm. Only anterior hooks with simple roots and no manubria. Lemnisci long, cylindrical, and slender………………………………..…….…*Profilicollis sphaerocephalus*

– Anterior trunk ovoid. Proboscis with 14–22 rows of 5–10 hooks each……………………………………… 5

5. In *Hemigrapsus crenulatus* from New Zealand. Proboscis with 14–16 rows of 5–8 hooks each reaching 85–110 μm. Hook tips recurved. Apical and posterior hooks rootless. Roots of larger anterior hooks simple, longer than blades. Lemnisci long, thick and cylindrical………………….……………….*Profilicollis novaezealandensis*

– Proboscis with more hook rows (15–22). Hook tips not recurved……………………………………………6

6. In *Neohelice* (=*Chasmagnathus*) *granulata* from Uruguay and Argentina. Proboscis with 16–22 rows of 7–9 hooks, each reaching 83–91 μm. Hooks similar but more slender posteriorly; only second anterior hook with short simple root. All other hooks slightly manubriated anteriorly with stubby roots. Lemnisci cylindrical, relatively longer than receptacle………………………………………………………*Profilicollis chasmagnathi*

– Proboscis hooks and roots dissimilar; hooks reaching 118 μm. Lemnisci variable………..………..….……7

7. In *Hemigrapsus crenulatus* & *Helice crassa* from Chile, New Zealand, South Shetland Islands. Proboscis with 18–22 rows of 6–9 hooks, each reaching 71–80 μm. Apical hook miniature. Anterior hooks with prominent simple roots and no manubria. Posterior hooks with abbreviated simple roots and well-developed manubria. Lemnisci cylindrical, thick, longer than receptacle, wider at middle…Profilicollis chasmagnathi*

– Proboscis with 15–22 rows of 7–10 hooks each. Shape of hooks, roots and lemnisci different………………8

8. Intermediate host unknown. In NW Canada and Russia. Proboscis with 22 rows of 7–8 hooks, each reaching 89–118 μm. Apical and basal hooks smallest, rootless. Anterior and middle hooks with simple roots. Posterior hooks more slender, gradually deceasing in size posteriorly, with rudimentary roots. Lemnisci pyriform, slightly longer than receptacle…………………………………………………………*Profilicollis arcticus*

– Proboscis with fewer hook rows (15–20) and different hook/root and lemnisci morphology……………..…9

9. In *Hemigrapsus oregonensis* and *Carcinus maenas* from North America, Scotland, and Eurasia. Proboscis with 15–20 rows of 7–8 hooks, each reaching 87–96 μm. Anterior and middle hooks with simple roots; posterior hooks with rudimentary roots. Lemnisci plump-bulbous, about as long as receptacle………………………………………………….…*Profilicollis botulus*

– In *Cancer irroratus* from Maine and from other intermediate hosts in Sweden and Europe. Proboscis with 16–20 rows of 7–10 hooks, each reaching 92–108 μm. All hooks without manubria and with simple roots except rootless basal hook. Lemnisci long, cylindrical, widest at middle……………..…*Profilicollis major*

* Second report of *Profilicollis chasmagnathi* from *Hemigrapsus crenulatus* was originally reported as *Profilicollis antarcticus.*

#### Description of cystacanths of *Profilicollis rancoensis* from *Aegla abtao* in Chile

**Figures 23–25:**
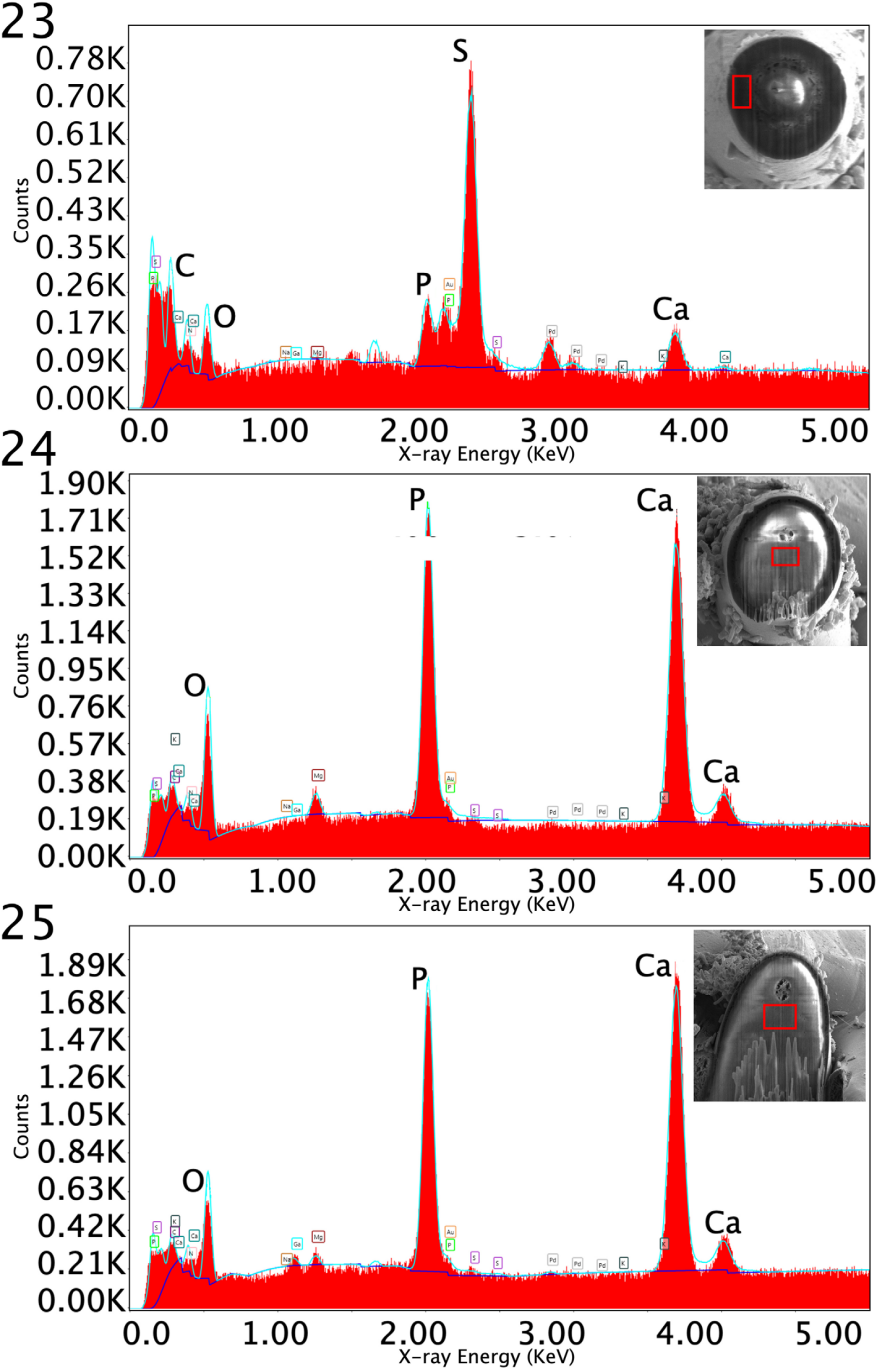
Energy-Dispersive X-ray Spectra (EDXS) of anterior hooks of *Profilicollis rancoensis* n. sp. cystacanths from *Aegla abtao* in Chile, acquired from the edge of a tip hook **(23)**, the center of the middle section of the hook **(24)**, and the center of the base of the hook (**25**), where the red inlays indicate the region from where the spectra were obtained. The spectra show the raw number of counts collected, and the elements C, O, P, S, Ca peaks highlighted. See Table 4 for EDXA numerical detail. Insets point to parts of hooks used to generate spectra.

With characters of the family Polymorphidae and genus *Profilicollis*. Body flattened dorso-ventrally, divided by constrictions into proboscis, long cylindrical neck, spinose anterior trunk, and ovoid posterior trunk (Figs. 1, 2). See comparative measurements and counts of species of *Profilicollis* in Table 2 and complete specific measurements of *P. rancoensis* in Table 3. Proboscis ovoid with blunt anterior end and 14–15 longitudinal rows of 6–7 hooks each. Hooks of similar shape (Fig. 3), gradually decreasing in length and diameter posteriorly from largest hooks (2nd to 4th). Basal hooks smallest with shortest roots and longest anterior manubrium. Some basal hooks appear to emanate from the neck and not directly from proboscis (Figs. 8, 10). All other hooks with slightly shorter roots and unremarkable anterior manubria (Fig. 3). Complete measurements of hooks in 1 longitudinal row in Table 3. Edge of hook tips with high level of sulfur and low levels of phosphorous and calcium and center of mid-hook showing an apparent opposite trend (Figs. 23, 24) and Table 4). Neck long widening at base. Proboscis receptacle with single-layered muscle wall extending from base of proboscis to about anterior third of spinose trunk. Primordia of cephalic ganglia detectable in receptacle just anterior to level of junction of neck with anterior trunk. Trunk with many electron-dense micropores (Figs. 21, 22) and many hypodermic nucleated cells appearing as round cysts. Anterior trunk with somewhat irregular circles of spines ending at trunk constriction. Spines pointed with broad base, dense cortical layer and spongy core (Fig. 4). Lemnisci long, slender, extending well beyond receptacle, often to level of posterior end of anterior trunk. In males, ovoid testes usually appear diagonally in anterior trunk (Fig. 1) but may also be less frequently found in posterior trunk or singly in both trunk segments at same time. One elemental tubular cement gland emerging from each testis posteriorly with rest of male reproductive system in posterior trunk remaining underdeveloped and undefined. Posterior trunk with corrugated texture on surface. In female cystacanths, parts of developing reproductive system barely distinguishable (Fig. 2). Inner subcutaneous muscle layer well developed in both trunk segments and fibrous connective tissue bundles join anterior and posterior trunks together (Fig. 1, 2).

**Table 4:** Elemental weight percent of Na, Mg, P, S, K, and Ca (as reported by TEAM EDXA software) of hooks and spines of cystacanths of *Profilicollis rancoensis* n. sp. from *Aegla abtao* in Chile, at 6 hook sites. Weight percentages of other elements (C, N, O, Al, Fe, Au, Pd, and Ga) are omitted.

Element*	Parts					
	Anterior hooks					
	Tip edge	Tip center	Middle center	Middle edge	Base edge	Base center
Sodium (Na)	**0.13** **	0.12	**0.02**	0.03	0.05	**0**
Magnesium (Mg)	**0.41**	1.16	**1.12**	0.46	0.35	**0.60**
Potassium (K)	**0**	0	**0**	0	0	**0.08**
Phosphorus (P)	**5.16**	16.75	**22.08**	8.79	8.49	**21.4**
Sulfur (S)	**27.78**	1.97	**0.02**	8.76	3.52	**0.19**
Calcium (Ca)	**7.56**	35.28	**46.67**	14.09	14.99	**49.67**
	Middle hooks					
Sodium (Na)	0.14	0.33	0.03	0.20	0.13	0.01
Magnesium (Mg)	0.26	0.85	1.89	0.98	1.03	1.30
Potassium (K)	0.14	0.27	0.08	0.10	0.04	0.13
Phosphorus (P)	2.67	9.90	22.48	10.57	12.57	20.72
Sulfur (S)	31.23	14.44	0.10	12.30	2.07	0.10
Calcium (Ca)	3.18	19.42	46.09	17.93	24.14	45.78
	Posterior hooks					
Sodium (Na)	0.12	0.12	0.05	0.05	0.02	0.06
Magnesium (Mg)	0.18	0.57	0.26	0.18	0.15	0.31
Potassium (K)	0.10	0.05	0.10	0.06	0.10	0.04
Phosphorus (P)	5.30	20.05	21.41	9.34	15.37	20.91
Sulfur (S)	17.70	1.16	0.25	9.34	2.69	0.10
Calcium (Ca)	11.91	45.70	50.93	17.95	39.11	51.27
	Whole anterior hook & longitudinal section of middle hook					
Sodium (Na)	0.04 & 0.02					
Magnesium (Mg)	0.17 & 1.73					
Potassium (K)	0 & 0.17					
Phosphorus (P)	4.39 & 23.73					
Sulfur (S)	3.59 & 0.18					
Calcium (Ca)	8.65 & 48.47					
	Spines (middle & longitudinal sections)					
Sodium (Na)	0.33 & 0.86					
Magnesium (Mg)	0.58 & 0.22					
Potassium (K)	0 & 0					
Phosphorus (P)	0 & 1.33					
Sulfur (S)	0.51 & 1.61					
Calcium (Ca)	0.87 & 0.62					

* Common protoplasmic elements (C, N, O) and processing elements (Au, Pd, Ga) are omitted.

** The bolded weight percentages of the anterior hooks are presented in Figure 5 (23–25).

#### Description of acanthellae of *Profilicollis rancoensis* from *Aegla abtao* in Chile

Our secondfourth collection of cystacanths on December 27 included two late acanthellae of slightly different ages measuring 2.25 × 0.75 and 2.75 × 0.6 mm, long × wide (Figs. 5, 6). The acanthellae were as large as the cystacanths and, like the cystacanths, were enveloped within membranous sheaths. Both acanthellae had conspicuously inverted invaginated proboscides with delicate sheaths and under-developed hooks. Single-layered proboscis receptacle prominent, with delicate sheaths, enclosing proboscides. Many mini nuclei reminiscent of the earlier giant hypodermal nuclei appeared in large number anteriorly in one specimen (Fig. 5). Each specimen had a developing or developed cercomer-like tail clearly absent in the cystacanths. The primordia (nuclei, fragments, or embryonic elements) of lemnisci, reproductive structures, trunk spines, cephalic ganglia or muscular layers in proboscis, receptacle or trunk were not evident.

#### Micropores

Micropores have been detected and documented from every single tissue studied including hooks (Fig. 18), proboscis (Fig. 19), spines (Fig. 20) and all regions of the trunk (Figs. 21, 22). It appears that every possible surface area in these cystacanths is dedicated to the differential absorption of nutrients that occurs through micropores.

#### Energy Dispersive X-Ray analysis (EDXA)

Energy Dispersive X-ray analysis was used to qualitatively assess the levels of various elements in the hard structures of the Acanthocephala, among other animals, to account for the hardness of structures such as the hooks and spines. As previously mentioned, EDX spectra were collected from the hooks at various surfaces exposed by the FIB milling, paying attention to the center (core) of the hook and the sheathing material (edge). Weight-percents for various metals (as reported by the TEAM software) are summarized in Table 4. Sample spectra and their typical collection areas are shown in Figure 23–25. Highest levels of sulfur were observed in the sheathing regions of the hooks (i.e., hook edges). The core regions of the hooks (i.e., base and center) contained mostly phosphorus and calcium, the two essential elements for hook structural support.

#### Molecular results

**Figure 26:**
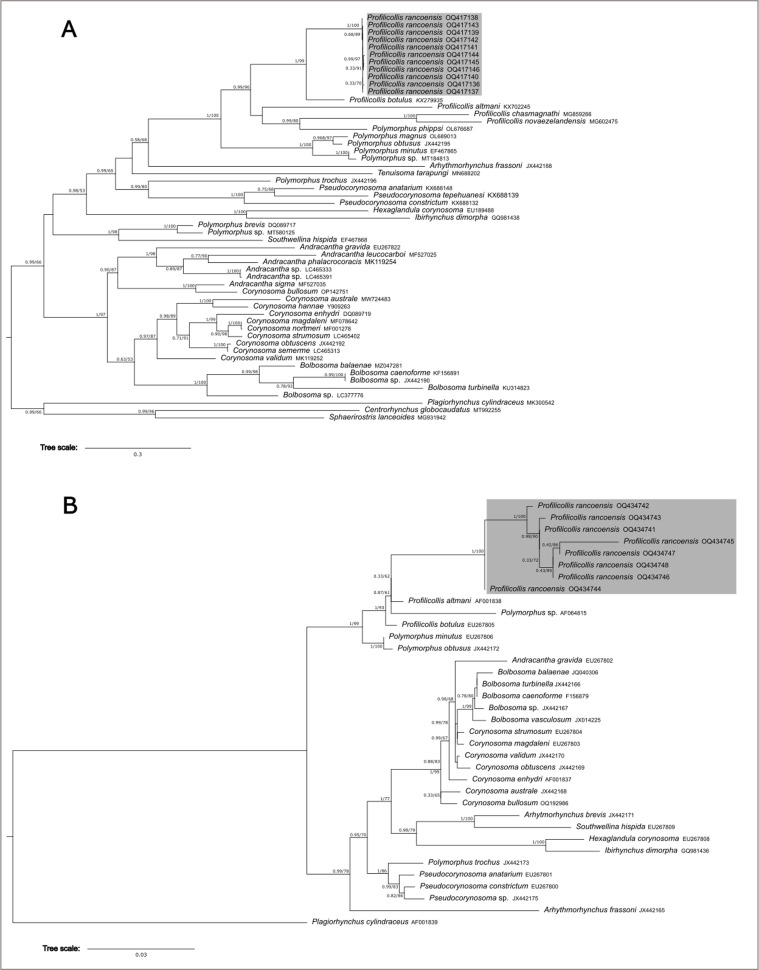
Genealogical relationships of haplotypes based on partial COI (A) and 18S (B) gene sequences of specimens of the family Polymorphidae recovered in a Bayesian inference analysis. Numbers next to nodes refer to support values. Bayesian posterior probability values are shown left, and Bootstrap proportions gathered in the Maximum Likelihood analysis (COI: L*n* = −11404.169, 18S: L*n* = −6647.679). GenBank accession numbers are included in the terminal labels.

**Figure 27:**
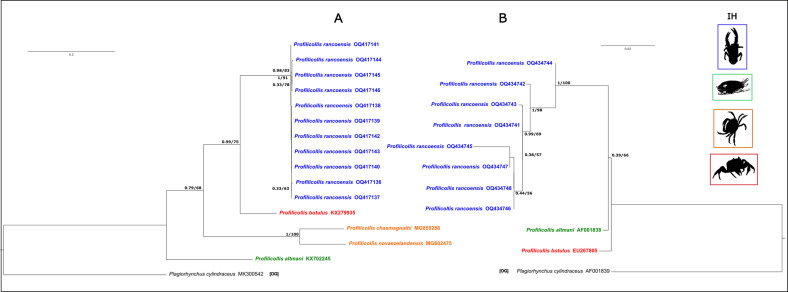
Genealogical relationships of haplotypes based on partial COI (A) and 18S (B) gene sequences of specimens of the genus *Profilicollis* recovered in a Bayesian inference analysis. Numbers next to nodes refer to support values. Bayesian posterior probability values are shown left, and Bootstrap proportions gathered in the Maximum Likelihood analysis (COI: L*n* = −2599.057, 18S: L*n* = −3574.388). GenBank accession numbers are included in the terminal labels. Animal silhouettes with different box colors to the right of the trees indicate the intermediate host (IH) of each species. Blue = *A. abtao*; Green = *Emerita* spp.; Orange = Varunidae; Red = Portunidae.

A total of 19 new sequences were successfully obtained (11 of the *COI* gene and 8 of the *18S* gene). These novel genetic sequences of *COI* and *18S* rDNA were 14.11% and 21.49% dissimilar, respective to those available in GenBank for *P. botulus*. Both phylogenetic trees gathered via ML and Bayesian inference (BI) for *COI* were congruent (Fig. 26A). The family Polymorphidae was recovered as shown to be monophyletic with high support only for the BI analysis and moderate support in the ML analysis (PP = 0.99; BS = 66). Within the clade of Polymorphidae, three main lineages were found (Fig. 26A). One lineage is a highly supported clade (PP = 1; BS = 97) and is formed by the genus *Andracantha, Corynosoma* and *Bolbosoma* (Fig. 26A). Within it, the genus *Andracantha* is found to be paraphyletic with respect to *Corynosoma bullosum*, while the other species of *Corynosoma* form a highly supported clade (PP = 0.97; BS = 89). The second main clade (PP = 1; BS 98) of Polymorphidae is formed by *Southwellina hispida* and a clade (PP = 1; BS = 100) formed by two of the three species of *Polymorphus* included in the analysis, *P. brevis* and *Polymorphus* sp. The last main lineage (PP = 0.98; BS 53) of Polymorphidae is formed by *Profilicollis, Polymorphus trochus, Arhytmorhynchus, Tenuisoma, Pseudocorynosoma, Hexaglandula,* and *Ibirhynchus*. Here, *Profilicollis* appears paraphyletic with respect to *Polymorphus phippsi*. This later species is sister, in a highly supported relationship (PP = 0.99; BS = 80), to the clade (PP = 1; BS = 100) formed by *Profilicollis chasmagnathi* and *P. novaezelandensis*. *Profilicollis botulus* is sister (PP = 1; BS = 99) to a highly supported clade (PP = 1; BS = 100) formed by all haplotypes of *P. rancoensis* (Fig. 27A).

The sample of *P. rancoensis* shows extremely low genetic variation. On average, the analyzed haplotypes of this species differed by 0.3%. For example, 3 specimens shared the same haplotype. Haplotypes of *P. rancoensis* differed from that of *P. botulus* by 15%. Similarly, *P. rancoensis* haplotypes differed by 24% and 28% from *P. chasmagnathi* and *P. novaezelandensis*, respectively.

The resulting ML and BI trees for the *18S* rDNA matrix are mostly congruent (Fig. 26B). The family Polymorphidae includes two lineages. One of these two lineages is formed by the genera *Andracantha, Bolbosoma, Corynosoma, Arhytmorhynchus, Southwellina, Hexaglandula, Ibirhynchus, Polymorphus*, and *Pseudocorynosoma*. This clade appears to be well supported, in particular in the IB analysis (PP = 0.99; BS = 78; Figs. 26A and 26B). The other main lineage is highly supported (PP = 1; BS = 99) and is formed by the genera *Profilicollis* and *Polymorphus*, which are not reciprocally monophyletic (Fig. 27B). Haplotypes of *P. rancoensis* form a highly supported clade (PP = 1; BS = 100). *Profilicollis rancoensis* forms a highly supported clade (PP = 1; BS = 93) with the species *P. altmani*, *Polymorphus* sp., and *P. botulus*. In turn, this clade is sister (PP = 1; BS = 99) to the clade (PP = 1; BS = 100) formed by *P. minutus* and *P. obtusus*, which fall in a strongly supported clade (Fig. 27B). The analyzed haplotypes of *P. rancoensis* differed on the average by 0.6%. In this case, two specimens shared the same haplotype. On average, haplotypes of *P. rancoensis* differed from those of *P. botulus* and of *P. altmani* by 3% and 2%, respectively.

## Discussion

### Concept and position of *Profilicollis* vs. *Polymorphus* based, in part, on Amin (1992)

The taxonomic status of *Profilicollis* has been in flux since Meyer [47] created the genus based on the long neck and spheroid proboscis and included 2 species, *P. botulus* and *P. arcticus.* The generic of features of *Polymorphus*, erected for one species, *P. minutus* (Goeze, 1782), by Lühe [42] included small body size, body wall nuclei, grid-like lacunar system, trunk spines anterior to constriction, radially symmetrical proboscis hooks which decreases in size anteriorly and posteriorly, double-walled proboscis receptacle with cephalic ganglion near its base or middle, long neck, moderately long lemnisci, testes behind one another, tubular cement glands, genital opening terminal without spines, and eggs with polar prolongation of middle membrane. Except for features like the long neck or the position of the testes, the above diagnosis remains largely valid today. As the number of species included in the genus increased, the generic concept continued to expand, *i.e.*, see Southwell and MacFie [66], Travassos [71], and Thapar [70]. Many of the diagnostic features added by various authors were too restrictive to be of generic value as the interspecific variability within *Polymorphus* continued to increase with the discovery of more species [1, 52]. These restrictive diagnostic features are noted in the following few paragraphs.

In Meyer’s [47] system, *Polymorphus* was correctly placed in his family Polymorphidae and order (Class) Palaeacanthocephala, and included 14 species [48] all of which are presently recognized members of the genus, except *P. magnus* which has been synonymized with *P. minutus.* In his brief diagnosis, Meyer [48] restrictively referred to short compact bodies cylindrical or weakly ovoid proboscides, trunk spines anterior to mid constriction, tubular cement glands, string-like lemnisci, and numerous small nuclei in the body wall. Meyer [47] also established a new genus, *Profilicollis*, for two long-necked species *Polymorphus botulus* and *Polymorphus arcticus* (=*Filicollis botulus* Van Cleave, 1916 and *Filicollis arcticus* Van Cleave, 1920) with eggs with concentric membranes, but kept *Filicollis* Lühe, 1911 for *Filicollis anatis* (Schrank, 1788) and *Filicollis sphaerocephalus* (Bremser in Rudolphi, 1819). Witenberg [80] and later Van Cleave [74–76] synonymized *Profilicollis* with *Polymorphus* and Van Cleave [74] reassigned *F. botulus* and *F. arcticus* as well as *F. sphaerocephalus* and *Filicollis altmani* Perry, 1942 to *Polymorphus* [76]. Webster [79] recognized the diversity within *Polymorphus* and erected a new subgenus, *Falsifilicollis* for forms with spheroidal proboscis and slender elongate neck previously included in *Filicollis*. These included *Polymorphus sphaerocephalus*, *Polymorphus altmani*, *Polymorphus kenti* Van Cleave, 1947 and *Polymorphus texensis* Webster, 1948. Schmidt and Kuntz [6591] correctly pointed out that *Profilicollis* Meyer, 1931 has priority for the concept of *Falsifilicollis* Webster, 1948. Petrochenko [52] erected Filicollidae for *Filicollis* Lühe, 1911 including only *F. anatis* (Schrank, 1788) Lühe, 1911, and *Parafilicollis* Petrochenko 1956 including *P. major*, *P. altmani*, *P. kenti* (a junior synonym of *P. altmani*) and *P. sphaerocephalus*. His three major diagnostic features of *Parafilicollis* were the long neck, spheroidal proboscis, and eggs without polar prolongation of middle membrane. Petrochenko [52] placed *Parafilicollis* in Gigantorhynchidae Southwell and MacFie, 1925 based primarily on the egg shape. Schmidt and Kuntz [65] declared *Parafilicollis* as without status because Meyer [47] erected *Profilicollis* for the same concept. The shape of eggs was clearly shown to be of no diagnostic value at the generic level in Polymorphidae by Van Cleave [75], Schmidt and Kuntz [64, 65] and Amin [1]. Golvan [24] accepted *Profilicollis* (= *Falsifilicollis* Webster, 1948) as a subgenus diagnosed with spheroidal proboscis and long slender neck, and which included the same four species listed by Webster [79], but without indicating a subgenus for the other species of the genus. Yamaguti [83] synonymized *Profilicollis* with *Polymorphus*, and *Parafilicollis* with Webster’s, 1948 *Falsifilicollis*, which he amended and elevated to the generic status and included *F. altmani*, *F. kenti*, *F. major*, *F. sphaerocephalus*, and *F. texensis*. Yamaguti’s [83] diagnosis of *Falsifilicollis* was essentially the same as that of Petrochenko’s [52] of *Parafilicollis* with few exceptions. *Falsifilicollis* and *Parafilicollis* clearly occupy the same concept for which Meyer [47] originally erected *Profilicollis*. Golvan [24] accepted *Profilicollis* with four species (*P. botulus*, *P. arcticus*, *P. altmani*, and *P. texensis*; a junior synonym of *P. altmani*) and recognized 26 species in *Polymorphus*. These included *P. kenti* and *P. major*, which, however, perfectly fit Golvan’s [24] own concept of *Profilicollis*; *Polymorphus* remained without an assigned subgenus. Hoklova [33] recognized *Profilicollis* as an independent genus with seven species, in Filicollidae; she had declared *Falsificollis* and *Parafilicollis* as synonyms. She did not, however, include *P. arcticus* (Van Cleave, 1920) Van Cleave, 1937 in her concept of *Profilicollis* but placed it in *Polymorphus* (*Polymorphus*) [32, 33, 73]. Her most complete diagnosis of *Polymorphus* was more encompassing of the wide diversity within the genus but still suffered from some of the restrictive characterizations stated by earlier observers. Nickol *et al*. [50] reached Hoklova’s [33] conclusions and “reintroduced” *Profilicollis* as a full-fledged genus after it had been recognized as a subgenus by many observers, including Schmidt and Kuntz [65] and Amin [2].

We update below the Amin [2] list of valid species of *Profilicollis* to show their synonymies and history.

**GENUS *Profilicollis* Meyer, 1931** [= *Falsifilicollis* Webster, 1948; *Parafilicollis* Petrochenko 1956]

1. *Profilicollis altmani* (Perry, 1942) Van Cleave, 1947 [= *Filicollis altmani* Perry, 1942; *Parafilicollis altmani* (Perry, 1942) Petrochenko, 1956; *Polymorphus bullocki* Mateo, Cordova, Guzman, 1982; *Profilicollis kenti* (Van Cleave, 1947) Hoklova, 1974; *Polymorphus kenti* Van Cleave, 1947; *Parafilicollis kenti* (Van Cleave, 1947) Petrochenko, 1956; *Falsificollis kenti* (Van Cleave, 1947) Yamaguti, 1963 fide Nickol et al. 2002; *Filicollis sphaerocephalus* sensu Harrington and Pillbury, 1938 fide Tantaleán et al. 2005; *Profilicollis texensis* (Webster, 1948) Hoklova, 1974; *Polymorphus* (*Falsificollis*) *texensis* (Webster, 1948) Yamaguti, 1963 fide Nickol et al. [50]].
2. *Profilicollis antarcticus* Zdzitowiecki, 1985.
3. *Profilicollis arcticus* (Van Cleave, 1920) Meyer, 1932 [= *Filicollis arcticus* Van Cleave, 1920].
4. *Profilicollis botulus* (Van Cleave, 1916) Witenberg, 1932 (type species) [= *Filicollis botulus* Van Cleave, 1916].
5. *Profilicollis chasmagnathi* (Holcman-Spector, Mane-Garzon, Dei-Cas, 1978) [1] [= *Falsifilicollis chasmagnathi* Holcman-Spector, Mane-Garzon, Dei-Cas, 1978].
6. *Profilicollis formosus* (Schmidt and Kuntz, 1967) [33] [= *Polymorphus formosus* Schmidt and Kuntz, 1967].
7. *Profilicollis major* (Lundström, 1942) [33] [= *Polymorphus major* Lundström, 1942; *Parafilicollis major* (Lundström, 1942) [74]; *Filicollis major* Lundström, 1942; *Falsificollis major* (Lundström, 1942) [83].
8. *Profilicollis novaezelandensis* Brockerhoff, Smales, 2002.
9. *Profilicollis rancoensis* n. sp.
10. *Profilicollis sphaerocephalus* (Bremser in Rudolphi, 1819) [76] [= *Echinorhynchus sphaerocephalus* Bremser in Rudolphi, 1819; *Filicollis sphaerocephalus* (Bremser in Rudolphi, 1819) [71]; *Parafilicollis sphaerocephalus* (Bremser in Rudolphi, 1819) [52]; *Falsifilicollis sphaerocephalus* (Bremser in Rudolphi, 1819) [83].

### Identification key

Our key, (above) is the only one designed from cystacanths even though some characters such as proboscis armature apply equally well to adults. All other published keys were created for adults and included such characters as size and shape of eggs, size of testes, and number of cement glands. Our key below is the latest and most complete.

### Hosts

The 87 extant species and subspecies of aeglid freshwater crabs restricted to South America are the only taxon within the infraorder Anomura (freshwater hermit crab *Clibanarius fonticola*) that has its life cycle entirely restricted to freshwater environments [61]. *Aegla abtao* is found in 410 km^2^ and 199 m deep Ranco Lake, the largest lake in the Los Rios Region, north Patagonia, Chile. The lake, once connected to the Pacific Ocean as an embayment, now lies between the Chilean Central Valley and the Andes, bordered by moraines around its western shore. The primary inflows of Ranco Lake are Calcurrupe, Caunahue, and Nilahue and the out flow is the Bueno River [14]. The crab is an omnivorous species in adults and there are records of its predatory behavior [38]. Irrespective of diet and food habits, crabs appear to become infected with acanthocephalan cystacanths by ingesting eggs that adhere to food sources (*e.g.*, plant debris), which are then deposited in the water body by definitive bird hosts [45, 55, 56].

### Micropores

The micropores of *P. rancoensis* n. sp. are associated with different proboscis and trunk regions corresponding to differential absorption of nutrients. We have reported micropores in a large number of acanthocephalan species [29] and in a few more since, and demonstrated tunneling from the cuticular surface into the internal crypts by TEM in *Corynosoma strumosum* (Rudolphi, 1802) Lühe, 1904 from the Caspian seal Pusa caspica (Gmelin) in the Caspian Sea (Figs. 19, 20 of Amin *et al*. [5]) and in *Neoechinorhynchus personatus* Tkach, Sarabeev, Shvetsova, 2014 from *Mugil cephalus* Linn. in Tunisia (Figs. 26, 29, 30 in Amin *et al*. [8]). Amin *et al*. [4] provided a summary of the structural-functional relationships of the micropores in various acanthocephalan species. Wright and Lumsden [82] and Byram and Fisher [15] reported that the peripheral canals of the micropores are continuous, with canalicular crypts constituting a huge increase in external surface area, implicated in nutrient uptake. Whitfield [81] estimated a 44-fold increase at a surface density of 15 invaginations per 1 μm^2^ of *M. moniliformis* tegumental surface.

### Energy Dispersive X-Ray analysis (EDXA)

Elemental composition of hooks and spines was evaluated via EDX spectra, with sulfur (S), calcium (Ca) and phosphorus (P) being the prominent elements in various species of acanthocephalans [28, 29]. In *P. rancoensis*, sulfur is usually prominent at the tip and the outer edge of large hooks and calcium and phosphorus are major constituents in the base and middle of the hooks, where tension and strength are paramount for hook function. Sulfur is part of the prominent outer layer of most acanthocephalan hooks and is a major contributor to the hardening process of hooks. While EDX spectra and weight-percentages are both regarded as highly qualitative, EDX analysis appears to be indicative of specific species. For example, sodium, a rarely prominent element, is evident in EDX spectra of whole hooks of *Microsentis wardae* (Martin and Multani, 1966) and of *Pallisentis nandai* Sarkar, 1953 [10], as well as in egg shells of *Neoechinorhynchus qatarensis* Amin, Saoud, Alkuwari, 2002 [28]. Similarly, *Moniliformis cryptosaudi* from Iraq is morphologically identical to *Moniliformis saudi* Amin, Heckmann, Mohammed, Evans, 2016 from Saudi Arabia, and it was erected based primarily on its distinctly different EDXA [7], as a cryptic species.

The elemental analysis of cystacanths of only 3 other species of acanthocephalans have previously been studied. In cystacanths of *Neoandracantha peruensis* (Amin, Heckmann, 2017) from the ghost crab in Peru, hooks near the middle of the proboscis showed comparatively very low sulfur (2.50%, 6.20%, 0.32% weight-percent as reported by TEAM) at the tip, middle, and base of the hook edge, and the corresponding levels of phosphorus and calcium were considerably higher (reaching 14.79% and 36.92%) at the base of the hook edge [3, 28]. This pattern is nearly opposite to that of *P. rancoensis* cystacanths and emphasizes the characteristic EDXA diagnosis for each species. Anterior and middle hooks of cystacanths of *Sphaerirostris picae* (Rudolphi, 1819) Golvan, 1956 from lizards and hedgehogs in Ukraine had moderate levels of phosphorus and sulfur (4.41–6.03%) and slightly higher levels of calcium (9.61–10.83%); again, showing variation in EDXA that is species specific [10]. Cystacanths of *Moniliformis kalahariensis* [Meyer, 1931], from *Blatella germanica* Linn. in India, had characteristically very low levels of phosphorus, sulfur, and calcium in anterior, middle, and posterior hooks alike [10]. A detailed discussion of the biological significance of EDXA as a diagnostic tool is exemplified by the observation that populations of an acanthocephalan species will consistently have similar EDX analysis irrespective of host species or geography, even though comparative morphometrics of different populations of the same species usually vary with host species and geography, as presented in Amin et al. [9, 10].

### Hosts and geography

Our molecular results corroborate that the cystacanth found in the freshwater crab *Aegla abtao* belongs to a new acanthocephalan species. The phylogenetic analysis showed that this new species falls into the genus *Profilicollis*, and the molecular analysis showed that *P. rancoensis* n. sp. exhibits a low level of genetic variation where recovered haplotypes differ on average 0.3% and 2% (COI and 18S, respectively). At the same time, *P. rancoensis* n. sp. is highly divergent from other species of *Profilicollis* included in the analysis (COI = 14% and 18S = 21% from *P. botulus*). These results are similar to those for the genus *Profilicollis* previously reported [9, 18, 54, 56], showing the influence of environmental variables on a specific host-parasite relationships, as well as that mobility of hosts is determinant in the strict parasite-intermediate host links [25, 41, 54, 56]. Regarding host habitat, environmental conditions may limit acanthocephalan species to particular crab hosts that are exposed to different habitats [41, 56, 67]. For example, *P. altmani* parasitized *Emerita* spp., while *P. chasmagnathi* inhabits varanid host, which belong to marine and estuarine environments, respectively [41, 54, 56]. In this study, *A. abtao* lives in freshwater environmental conditions, becoming the first *Profilicollis* species that lives in this host species and in these systems in South America. On the other hand, birds belonging to the order Charadriiformes have been reported as definitive hosts for parasites of this genus. Unfortunately, in our fieldwork, we did not find adult worms or the definitive host, although species such as kelp gulls *Larus dominicanus*, brown-hooded gulls *Chroicocephalus maculipennis*, and cormorants *Phalacrocorax olivaceus* (Order Suliformes) are typical inhabitants of these habitats, and crabs are key prey in their diet [41, 56]. Therefore, close parasite-intermediate host relationships are often ascribed to specific environmental conditions that vary among host species [41, 56, 67].

In general, there are several taxa in Polymorphidae that do not form monophyletic groups, such the genus *Profilicollis* [6, 9, 41, 54, 56]. These results indicate that the systematic positions of the Acanthocephala are still unstable, and the composition of the genera need to be revised. Although most groups have been proposed on the basis of morphological and morphometric characters, they need to be supported by an explicit phylogenetic approach [9, 17, 20, 52].

## Figures and Tables

**Figure S1:**
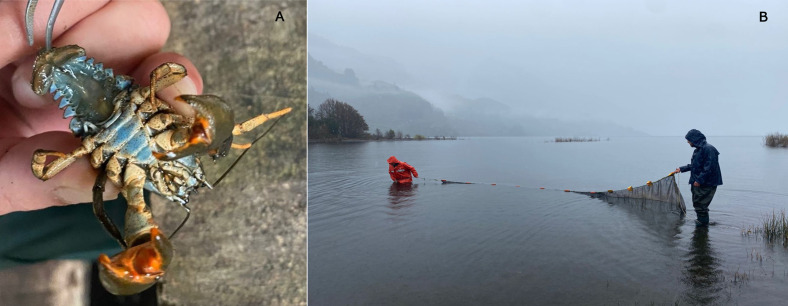
(A) Image of individual of the freshwater crab *Aegla abtao*. (B) = Field work and collection of *A. abtao* in Ranco Lake, northern Patagonia, Chile.

## Supplementary material

The Supplementary materials of this article are available at https://www.parasite-journal.org/10.1051/parasite/2023042/olm.
